# Collision with opponents—but not foul play—dominates injury mechanism in professional men’s basketball

**DOI:** 10.1186/s13102-021-00322-z

**Published:** 2021-08-19

**Authors:** Leonard Achenbach, Christian Klein, Patrick Luig, Hendrik Bloch, Dominik Schneider, Kai Fehske

**Affiliations:** 1 Department of Orthopedics, König-Ludwig-Haus, Julius-Maximilians-University Würzburg, Brettreichstraße 11, 97074 Würzburg, Germany; 2 Department of Trauma, Hand, Plastic and Reconstructive Surgery, University Medical Center Würzburg, Würzburg, Germany; 3 German Statutory Accident Insurance for the Administrative Sector (Department of Sports Injury Prevention), VBG, Hamburg, Germany; 4 Faculty of Medicine, University Würzburg, Würzburg, Germany

**Keywords:** Epidemiology, Mechanism, Contact, Non-contact, Injury prevention, Match load

## Abstract

**Background:**

To identify injury patterns and mechanisms in professional men’s basketball by means of video match analysis.

**Methods:**

In Germany, injuries are registered with the statutory accident insurance for professional athletes (VBG) by clubs or club physicians as part of occupational accident reporting. Moderate and severe injuries (absence of > 7 days) sustained during basketball competition in one of four seasons (2014–2017 and 2018–2019) in the first or second national men’s league in Germany were prospectively analyzed using a newly developed standardized observation form. Season 2017–2018 was excluded because of missing video material.

**Results:**

Video analysis included 175 (53%) of 329 moderate and severe match injuries. Contact patterns categorized according to the different body sites yielded eight groups of typical injury patterns: one each for the head, shoulders, and ankles, two for the thighs, and three for the knees. Injuries to the head (92%), ankles (76%), shoulders (70%), knees (47%), and thighs (32%) were mainly caused by direct contact. The injury proportion of foul play was 19%. Most injuries (61%) occurred in the central zone below the basket. More injuries occurred during the second (OR 1.8, *p* = 0.018) and fourth quarter (OR 1.8, *p* = 0.022) than during the first and third quarter of the match.

**Conclusion:**

The eight identified injury patterns differed substantially in their mechanisms. Moderate and severe match injuries to the head, shoulders, knees, and ankles were mainly caused by collision with opponents and teammates. Thus, stricter rule enforcement is unlikely to facilitate safer match play.

## Background

Basketball is associated with a high risk of injury, particularly in professional league settings [1]. Injury prevention requires the precise analysis of situations resulting in injury to understand the mechanisms and causes of acute injuries [2–4]. Full understanding of injury mechanisms is only possible in a sports-specific context. Yet, little is known about the match situations and the behavior of players and their opponents at the time of injury. Knowledge of the injury mechanisms in professional men’s basketball may help to establish more targeted injury prevention measures [3, 4]. Here, valuable data are obtained from video match analyses of injury situations in team sports [5–9].

The situations leading to injury in basketball and the mechanisms resulting in the different types of injury are not well documented. Previous studies on basketball only focused on injuries to the Achilles’ tendon [10], the ankle joint [11], or the anterior cruciate ligament in female athletes [12–15]. Thus, the purpose of this explorative study was to identify patterns of situations and mechanisms leading to moderate and severe injuries in professional men’s basketball in a league setting by means of video match analysis.

## Methods

This prospective study was conducted over the seasons 2014–2015, 2015–2016, 2016–2017, and 2018–2019. Video material was missing for season 2017 to 2018 was excluded. This study included all male basketball players of the first and second national league in Germany who had played in at least one match in the above seasons. Injuries and contact mechanisms were defined by video match analysis in team ball sports as described previously [5, 6]. Inclusion and exclusion criteria, procedure of video production, provision of video footage, data collection, video analysis, and the concordance of referee decisions with the analyses of an expert video rater were identical to those described in previous literature reports [5, 6].

### Development of the observation form

The observation form, which was specifically designed for basketball, followed the latest versions of already established surveillance forms in other team ball sports [5, 6] and contained 25 factors in 5-item groups (Table 1). In a pilot test of this version, 9 selected injury sequences (3 injuries each to the head, knees, and ankles) were evaluated by 10 naive raters experienced in basketball [16]. Using Cohens measures and Fleiss-Kappa (κ) measures for bi-rater and multi-rater analyses, the analysis of inter-reliability for the final observation form resulted in very good concordance (κ = 0.85, range 0.51–1.00). Five items showed perfect agreement (κ = 1.00), 8 items substantial agreement (range 0.64–0.78), and 2 items (ball possession, own foul) moderate agreement (range 0.51–0.58). The observation form yielded very good concordance for intra-rater reliability (κ = 0.92, range 0.79–1.00).

**Table 1:** Item groups, categories, and factors of the observation form for basketball

Item group	Category	Factor
I	General	Identification code of injury, club, date of match, type of competition (league match, cup match), number of match (e.g. league match), type of match (home, away), match minute of injury, playing position of injured player (point guard (PG), shooting guard (SG), small forward (SF), power forward (PF), center (C))
II	Court area	7 frontcourt and 7 backcourt zones
III	Ball possession	Injured player, own team, direct opponent, opponent team, none
	Game action	General: standing, starting, running, sprinting, side-step, stopping, change-of-direction, taking-off, being mid-air, landing
		Basketball-specific: passing, lay-up/dunking, catching, penetration, shooting, blocking, screening/picking, closing out, boxing out, rebounding, other 1-on-1 situation, faking, fighting for the ball, screen defense, posting up, help defense, other
	Game phase	Set offense, fast break, transition defense, halfcourt defense, securing the ball, other
	Floor contact	Both legs, single leg, no contact
	Interaction with other players	No other player, teammate, opponent, both
	Foul play subjective*	No foul, foul play by opponent, foul play by teammate
	Referee decision	No foul, foul play by opponent, foul play by injured player
		No penalization, defensive foul, unsportsmanlike foul, disqualifying foul
VI	Main mechanism	Contact, indirect contact, non-contact
	Detailed mechanism	(a) Collision with opponent, collision with teammate, collision with ball, other collision, hit/push of opponent, pull/hold of opponent, other interaction with opponent
		(b) Fall, ankle twist, knee twist, slip, overload, other
V	Injured body site	Head, neck, shoulder, upper arm, elbow, lower arm, wrist/hand, finger, trunk, hip, thigh, knee, lower leg, ankle/foot, unidentified
	Initial contact with player's body site*	Head, neck, shoulder, upper arm, elbow, lower arm, wrist/hand, finger, trunk, hip, thigh, knee, lower leg, ankle/foot, unidentified
	Initial contact with body site of opponent*	Head, neck, shoulder, upper arm, elbow, lower arm, wrist/hand, finger, trunk, hip, thigh, knee, lower leg, ankle/foot, unidentified

*Optional

### Video analysis

One expert rater (DS, former professional basketball player and physician) reviewed and classified each incident according to the observation form. A detailed description of the video analysis process is given elsewhere [5, 6]. Typical injury patterns were described for each body site. Inclusion criteria were a sufficient frequency of the respective type of injury and that the injury was caused by one of the three contact mechanisms (contact, indirect contact, or non-contact). Inclusion of an injury into the analysis was based on a consensus between all authors.

### Statistical analysis

The main injury mechanism, the different types of injuries, and injury severity were analyzed with X^2^ tests and the Fisher exact test. X^2^ tests were used to compare injury proportions between the four match quarters. Odds ratios (OR) and 95% CI are reported. Agreement of the video rater with referee decisions was quantified with Cohens measures and Fleiss-Kappa (κ) measures. The significance level was set to *p* < 0.05. All analyses were conducted using IBM SPSS Statistics, version 24.0.

## Results

This analysis included 175 (53%) of 329 identified moderate and severe match injuries (Table 2): 173 (99%) injuries were sustained during matches in either of the two national leagues and 2 (1%) during the German Cup. Point guards (n = 46, 26%) sustained more injuries than shooting guards (n = 36, 21%), power forwards (n = 36, 21%), center players (n = 34, 19%), or small forwards (n = 23, 13%). The body sites most often affected by injury were the ankles (n = 59, 34%), knees (n = 34, 19%), and thighs (n = 19, 11%). Compared to the total number of injuries, head and ankle injuries were slightly overrepresented in the video dataset, whereas shoulder, hip/groin, and lower leg injuries were slightly underrepresented.

**Table 2:** Selected and identified injuries for each season

Season	Number of players n	All registered injuries n	All registered moderate and severe match injuries n	Identified match injuries n %
2014–2015	514	1045	70	47 (67%)
2015–2016	522	994	73	41 (56%)
2016–2017	508	1008	80	43 (54%)
2018–2019	509	923	106	44 (43%)
Total*	871	3970	329	175 (53%)

*The number of players is duplicated for each season

### Match time and field area of injury occurrence

**Fig. 1:**
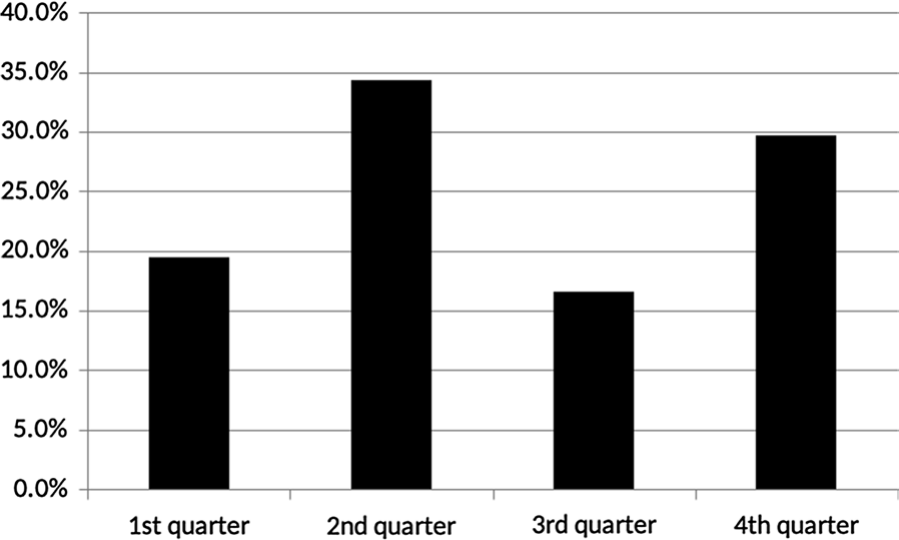
Distribution of match injuries in men’s basketball divided by quarters

Of the 175 injuries analyzed, 62 (35%) injuries were sustained during set offense, 56 (32%) during halfcourt defense, 19 (11%) during fast breaks, 18 (10%) while securing the ball, and 15 (9%) during transition defense. No injuries were sustained during overtime. 5 (3%) injuries were classified as other. Injuries were similarly distributed between the first (54%) and second match half (46%, *p* = 0.35). The four match quarters differed in injury distribution (Fig. 1): The number of injuries in the second quarter was higher than in the first quarter (OR 1.8, 95% CI 1.1–2.8, *p* = 0.0179) and higher in the fourth quarter than in the third quarter (OR 1.8, 95% CI 1.0–3.0, *p* = 0.022).

**Fig. 2:**
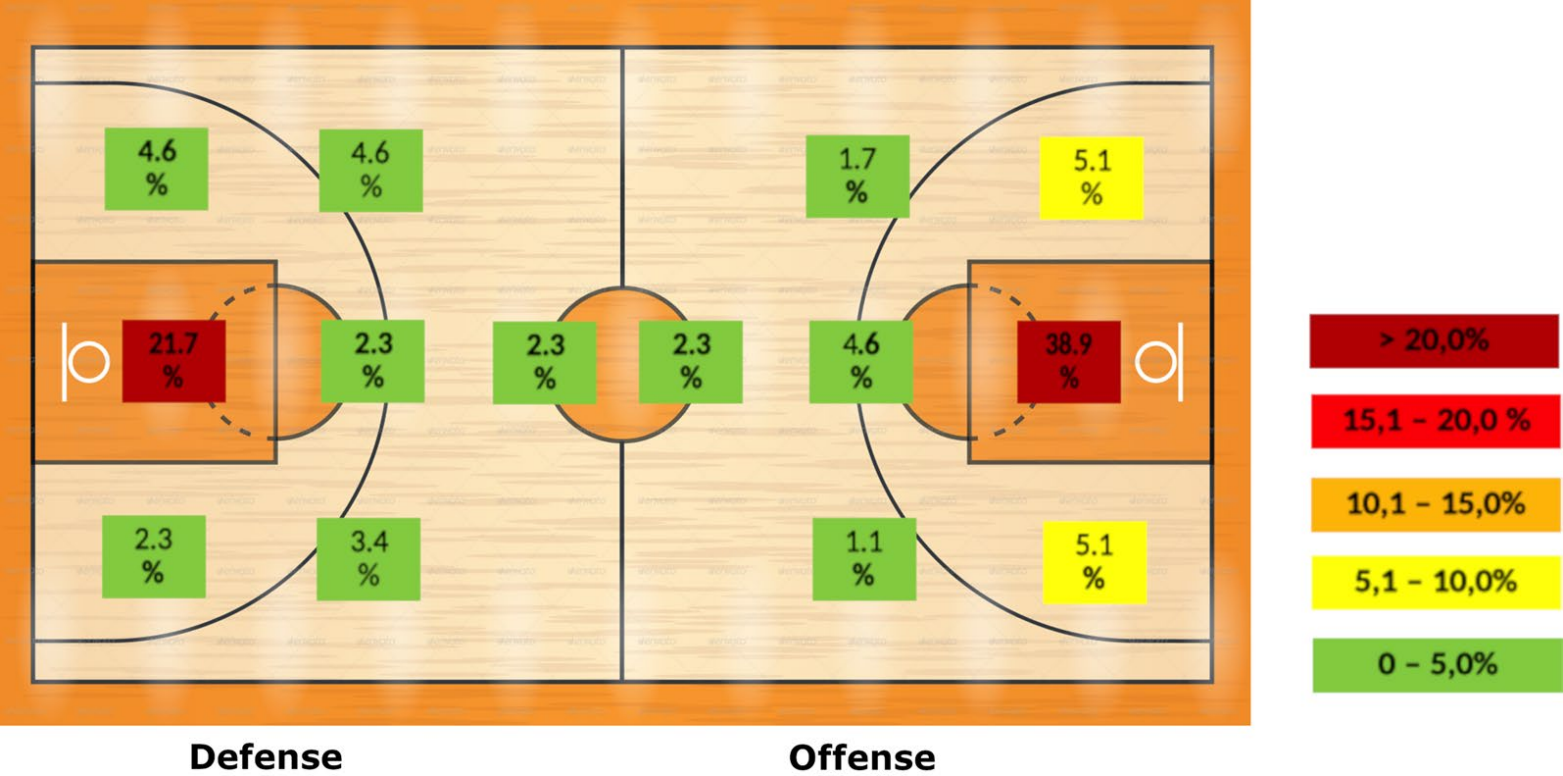
Distribution of match injuries in men’s basketball divided by field areas. The left part shows the defense area and the right part the offense area

Of all injuries analyzed, 103 (59%) were sustained in the frontcourt and 72 (41%) in the backcourt. Most injuries (n = 106 injuries, 61%) occurred in the central zone below the basket (Fig. 2).

### General and basketball-specific movements

Landing (n = 67, 38%) and running (n = 42, 24%) were the most frequent general movements, less frequent were standing (n = 13, 7%), jumping (n = 11, 6%), stopping (n = 10, 6%), change-of-direction movements, side-steps (both n = 9, 5%), being mid-air (n = 8, 5%), sprinting (n = 4; 2%), and starting (n = 2, 1%).

The most frequent basketball-specific movement patterns were rebounding (n = 25, 14%), layup or dunking (n = 24, 14%), other 1-on-1 situations (n = 23, 13%), actions classified as other (n = 17, 10%), shot blocking (n = 16, 9%), shooting (n = 14, 8%), boxing out, screen defense (both n = 11, 6%), penetration (n = 8, 5%), passing (n = 7, 4%), fighting for the ball (n = 6, 3%), catching, posting up (both n = 3, 2%), help defense, closing out, faking (n = 2, 1% each), and screening (n = 1, 1%).

### Contact mechanisms

**Fig. 3:**
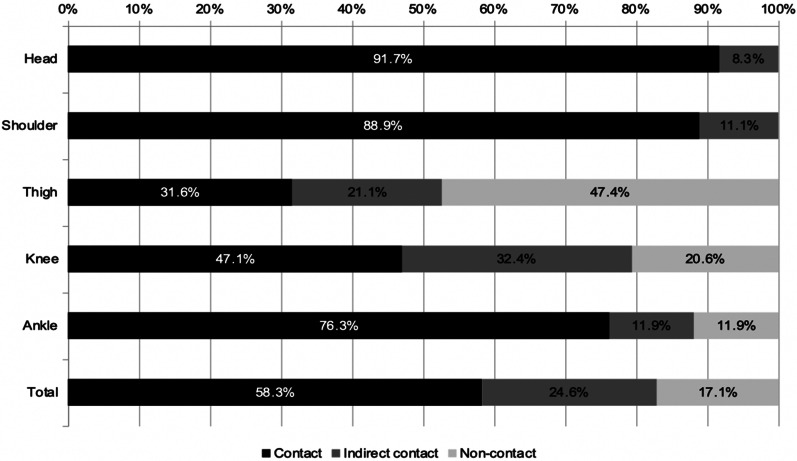
Proportion of contact mechanisms for the most frequently injured body sites during match play in men’s basketball. Contact mechanisms are divided into contact (black), indirect contact (dark gray), and non-contact (light gray)

**Fig. 4:**
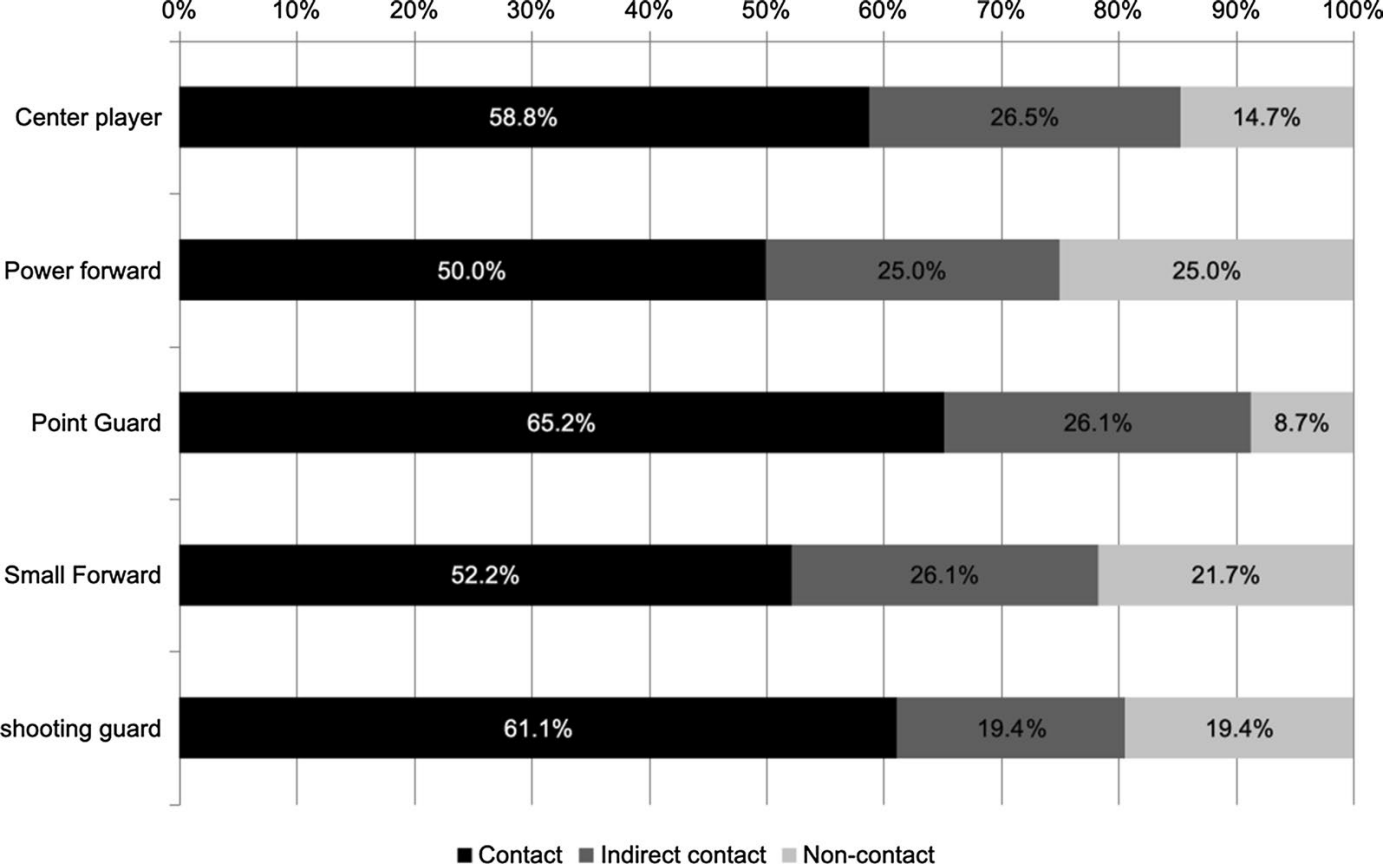
Proportion of contact mechanisms for the five field positions during match play in men’s basketball. Contact mechanisms are divided into contact (black), indirect contact (dark gray), and non-contact (light gray)

Head, shoulder, and ankle injuries were mainly sustained through direct contact (all *p* < 0.0001). In contrast, more than two thirds of injuries to the thighs were non-contact injuries, and one third of knee injuries detected in the video recordings had occurred due to indirect contact (Fig. 3). The proportion of contact injury mechanisms also differed between the four playing positions (Fig. 4). Point guards sustained the highest number of contact injuries (n = 30, 65%, *p* = 0.28) and power forwards the highest number of non-contact injuries (n = 9, 25%, *p* = 0.16) compared to the average of the other positions. Indirect contact injuries were equally distributed, ranging from 19 to 26%.

The most frequent mechanism of the 102 (59%) contact injuries was collision with an opponent (n = 69, 68%), 35 (51%) of which were caused by foot-to-foot contact. Less frequent mechanisms were being pushed or hit by an opponent (n = 14, 14%, 7 elbow-to-head) or collision with a teammate (n = 12, 12%, 7 foot-to-foot).

43 (25%) indirect contact injuries were also caused by collision with an opponent (n = 31, 72%) resulting in a twist (n = 9, 29%) or fall (n = 8, 26%). Other frequent injuries were sustained due to overload after collision with an opponent (n = 7, 23%).

Most of the 30 (17%) non-contact injuries were due to overload (n = 15, 50%), mainly of the thigh (n = 8), and twisting the ankle (n = 9, 30%). Injuries due to twisting the knee were less frequent (n = 3, 10%).

### Foul play and ball possession

Of all injuries, 101 (58%) were sustained in match situations involving the ball. 33 (19%) injuries were associated with foul play: 21 (64%) by the opponent and 12 (36%) by the injured player himself. These injuries were rated by the referees as defensive fouls (28), offensive fouls (2), or unsportsmanlike fouls (3). The expert rater agreed with the referee decisions in 93.1% (95% CI 0.89–0.96), which resulted in good concordance (κ = 0.79, 95% CI 0.68–0.90**)*.***

### Typical injury patterns

Overall, eight typical injury patterns of moderate and severe match injuries in professional men’s basketball were identified: one each for the head, shoulders, and ankles, two for the thighs, and three for the knees. The patterns differed considerably in their mechanisms and causes (Table 3). 2 of 9 injury patterns were discussed, of which one was finally excluded by consensus among all authors.

**Table 3:** Most common repetitive injury patterns in professional men’s basketball

Injury pattern	Injured body site	Frequency of pattern: n/n total (%)	Main mechanism: frequency within pattern (n/n total of pattern)	Common injury types: frequency within pattern (n/n total of pattern)	Foul play: frequency within pattern (n/n total of pattern)	Detailed mechanism: frequency within pattern (n/n total of pattern)	Movement pattern: frequency within pattern (n/n total of pattern)	
							Basic	Basketball-specific
(1) ‘Elbow-to-head injury’	Head	11/12 (92%)	Contact	Concussion (5/11), fracture (4/11), mainly of nasal septum or nasal bone (3/4)	Sometimes (fouled 2/11; own foul 1/11)	Hit or being pushed by an opponent (9/11), mainly by elbow-to-head contact (7/9)	Standing (3/11), being mid-air (3/11)	Rebounding (4/11)
(2) ‘Shoulder collision injury’	Shoulder	7/10 (70%)	Contact	AC joint separation (4/7)	Rare (fouled 1/7; own foul 0/7)	Collision with opponent (4/7), often with shoulder-to-shoulder contact (3/4)	Running (5/7)	Screen defense (4/7)
(3) ‘Overload thigh muscle injury’	Thigh	9/19 (47%)	Non-contact	Muscle tear (6/9)	No	Overload (8/9)	Sprinting (2/9), stopping (2/9)	Other 1 on 1 situation (4/9), penetration (3/9)
(4) ‘Knee-to-thigh muscle injury’	Thigh	6/19 (32%)	Contact	Muscle tear (3/6), contusion (3/6)	Rare (fouled 1/6; own foul 0/16)	Collision with opponent (4/6), mainly by knee-to-thigh contact (3/4); hit or being pushed by an opponent with knee-to-thigh-contact (2/6)	Running (2/6)	Other 1 on 1 situation (2/6)
(5) ‘Collision-with-opponent knee injury’	Knee	16/34 (47%)	Contact	(Partial) rupture of knee ligaments (8/16), mainly of the MCL (5/8)	Rare (fouled 0/16; own foul 2/16)	Collision with opponent (12/16), mainly by knee-to-knee contact (4/12); collision with teammate (4/16), mainly by knee-to-trunk contact (2/4)	Standing (6/16), landing (5/16)	Rebounding (4/16)
(6) ‘Collision-and-twist knee injury’	Knee	11/34 (32%)	Indirect contact	(Partial) rupture of knee ligaments (5/11)	Sometimes (fouled 1/11; own foul 2/11)	Collision with opponent (10/11), resulting in twisting of the knee (7/10)	Landing (4/11),side-step (3/11)	Other 1 on 1 situation (5/11)
(7) ‘Non-contact knee injury’	Knee	7/34 (21%)	Non-contact	(Partial) rupture of knee ligaments (4/7), mainly of the MCL (2/4) and the ACL (2/4)	No	Twisting of the knee (3/7), overload (3/7)	Change of direction (3/7), jumping (2/7)	Rebounding (2/7), other 1 on 1 situation (2/7)
(8) ‘Foot-to-foot ankle injury’	Ankle	45/59 (76%)	Contact	Sprain or partial/full tear of medial or lateral ligaments (41/45)	Rare (fouled, 5/45; own foul, 1/45)	Collision with opponent with foot-to-foot contact (33/45), collision with teammate with foot-to-foot contact (7/45)	Landing (25/45), running (11/45)	Rebounding (9/45), lay up or dunking (7/45)

Foul play was classified as 0% = no, 1–25% = rare, 26–50% = sometimes, 51–75% = common, and 76–100% = very common

*ACL* anterior cruciate ligament, *PCL* posterior cruciate ligament, *MCL* medial collateral ligament, *AC joint* acromioclavicular joint

## Discussion

The most important finding of this study was the identification of eight typical injury patterns of acute, moderate, and severe injuries in professional men’s basketball. Particularly collisions could be identified as one of the main mechanisms of injuries to the head, shoulders, thighs, knees, and ankles. The identified typical basic, sports-specific movement patterns and contact situations, as well as the main injury mechanisms may provide valuable data for investigating future preventive approaches in professional men’s basketball.

Typical movement patterns were running with quick change-of-direction movements, sharp cutting movements, jumping, and landing. Of all moderate and severe injuries sustained in a professional men’s basketball league setting, 58% were caused by direct contact, 25% by indirect contact, and 17% by non-contact mechanisms. Contact injuries often occurred when the athlete was mid-air. Indirect contact injuries were sustained during shooting and landing, whereas non-contact injuries occurred during faking, especially during change-of-direction movements and sprints. The detailed mechanisms of moderate and severe injuries in professional men’s basketball in a league setting differed for each body site.

### Injury mechanisms

Of the eight identified mechanisms of moderate and severe injuries, six directly or indirectly involved collision with another body part, mainly of an opponent.

In our study, head injuries occurred after a hit or push by an opponent, mainly through direct elbow-to-head collisions. Our findings extend findings from other team ball sports regarding a major number of contact mechanisms in moderate and severe head injuries [5, 6, 17–19].

Collision with an opponent played an important role in most injuries to the shoulders, thighs, knees, and ankles. Moderate and severe shoulder injuries often occurred by shoulder collision with an opponent, mainly shoulder-to-shoulder collision during 2-on-2 situations. In contrast, injuries due to thigh collision were mainly sustained through direct knee-to-thigh contact in various match situations.

Ankle sprain is generally considered to be the one of the most common sports-specific injury in basketball [1, 20, 21]. The consistent feature in this injury pattern is sudden inversion and slight plantar flexion, with or without internal rotation [11]. We identified a highly repetitive mechanism of moderate and severe injuries resulting from direct contact, mainly foot-to-foot collision with the foot of an opponent or a teammate after landing from a rebound or a jump shot: 45 out of 59 analyzed ankle injuries matched this pattern. This result confirmed the findings of previous studies on indoor team ball sports, such as handball [5].

Although direct collision was identified as the main injury feature in the majority of injuries, knee injuries were sustained due to different contact mechanisms. Injury patterns differed substantially in mechanisms, corresponding to previous findings in basketball [22]. Of all identified moderate and severe knee injuries in professional men’s basketball, 21% resulted from non-contact knee twisting without any external impact. Our findings confirmed the basic and basketball-specific movement patterns, including landing and change of direction as known from other team ball sports [5, 6]. The identified injury patterns extend previous findings on the patterns of indirect and direct contact injuries. More than one third of all moderate and severe knee injuries were sustained due to indirect contact, for instance, twisting of the knee mainly occurred after a collision that may have disrupted the initial movement pattern and thereby facilitated injury. The frequently observed direct external impact often resulted from collision with opponents or teammates.

Our findings on thigh injuries confirmed the results of other team ball sports regarding the number of non-contact mechanisms [5, 6, 23, 24]. Almost 50% of all thigh injuries were non-contact injuries sustained during sprints and running. None of the identified injuries had been sustained because of foul play. Based on the findings of the described injury patterns, thigh injuries in professional men’s basketball are primarily caused by peaks of high load.

### Foul play and relevance for refereeing

The results of our study showed that foul play is not a key injury mechanism in professional men’s basketball in a league setting. The proportion of 19% of moderate or severe injuries sustained in a league setting because of foul play was low and even less when only counting foul play by opponents (12%). These rates are lower than those found in other team ball sports, such as football (soccer) (22%) [6] or handball (28%) [5], and can be explained by the stricter contact rules in basketball than in football or handball.

With the possible exception of head injuries, rule changes and stricter refereeing in professional men’s basketball in a league setting does therefore not translate to safer match play. Rule reinforcements have proven to be an appropriate approach to reduce head injuries in distinct match situations in football but may not necessarily result in lower injury rates in professional men’s basketball. In football, referees have been instructed to severely sanction fouls involving the use of arms in tackles during vertical jumps because this type of foul has been identified as the most frequent cause of severe head injury [25]. Such stricter refereeing resulted in a 48% decrease in the number of head injuries between the 2002 and the 2006 FIFA World Cups [19] and in a 30% decrease in Bundesliga matches between the seasons 2000–2001 to 2005–2006 and the seasons 2007–2008 to 2012–2013 [24]. This reduction has probably been—at least in part—caused by stricter refereeing rules [26]. Thus, strict implementation of the rules by referees may potentially help to maintain the low number of head injuries due to foul play in professional men’s basketball. However, because of the different character of match play and injury patterns in basketball, the degree of reduction may not be as effective as in football and should thus be interpreted with caution.

### Field area, match phase, and time of injury

Basketball is a game characterized by a high number of transitions, i.e. continuous switches between defensive and offensive play. Most injuries (35%) were sustained during set offense. The field area with the highest number (61%) of moderate and severe match injuries was the central zone below the basket [27, 28]. This finding was not unexpected because this zone is marked by high-intensity play and players attempt to score often. Thus, basketball has a higher proportion of injuries in one specific field area than other team ball sports, such as handball in which the respective rate is about one third [5].

Although the number of injuries sustained in each of the two half times of a match was similar, the proportion of injury increased in the last quarter of each match half. Potential causative mechanisms such as players’ substitution or fatigue should be further analyzed for their potential of injury prevention.

### Relevance for injury prevention in daily practice

Most of the identified injury mechanisms of moderate and severe injuries involved collision with an opponent or teammate. Injuries due to direct collision can never be completely excluded in fast-paced ball sports. Yet, training and match preparation may be improved by preparing athletes for indirect contact during passing and lay-up and for direct contact during jumps, change-of-direction movements, and landing. The identified patterns of indirect contact injuries propose the examination of injury prevention measures for improving athletes’ ability to withstand upper-body perturbation without any subsequent uncontrollable twisting of the knees.

In addition to these direct and indirect contact injuries, the knees and thighs were shown to be susceptible to non-contact injuries. Non-contact knee and thigh injuries are often associated with intrinsic risk factors. Addressing such intrinsic (neuromuscular) risk factors has been shown to be effective in lowering injury risks, thereby reducing acute severe knee injuries, such as ACL tears or hamstring injuries, in team ball sports by at least 50% [29–31]. Training programs for improving muscular strength [32, 33] and training load measures [34] should be evaluated for their effectiveness in reducing (non-contact) injuries in professional men’s basketball.

Together, these findings have huge preventive potential. Multifaceted preventive approaches are needed that focus on respective basic and sports-specific movement patterns, contact situations, and the main (contact-) mechanisms of situations leading to injury.

## Strengths and limitations

The strength of this study is its prospective data collection in two professional national men’s basketball leagues over four seasons. To ensure high-validity data, this study used insurance data directly registered by team physicians [35, 36].

The main limitation is the low identification rate of 53%. The main reasons for non-identification in this study can be found in previous publications [5, 6, 9], but our rates compare to the rates of 30–54% of other research in professional team sports [5–7, 24, 37, 38]. It is important to note that our data do not apply to every acute moderate and severe injury in professional men’s basketball and that our data may be biased towards direct and indirect contact injuries. Especially head injuries were assumed to be overrepresented compared to other body sites because of easier identification in match situations due to interruption of the match [9]. Finally, video analysis is not an accurate method for the etiological description of overuse injuries because such injuries cannot be attributed to a single inciting event. Thus, the present study was only focused on acute injuries. Systematic bias due to the final observation by only 1 rater cannot be excluded.

## Conclusion

Collision with opponents and teammates was the predominant for moderate and severe match injuries to the head, shoulders, knee, and ankles in professional men’s basketball in a league setting. Eight typical injury patterns for moderate and severe injuries could be identified. Performance-determining techniques of 1-on-1 or 2-on-2 with direct and indirect contact are associated with a high risk of injury and should thus be part of routine training programs. Adequate jumping and landing skills as well as change-of-direction movements with intense physical contact are typical for basketball and should be a central component of technical training. Only a low proportion of injuries in professional men’s basketball in a league setting was associated with foul play. Thus, stricter rule enforcement does not seem to translate to safer match play.

## Data Availability

Data may have been obtained from a third party and are not publicly available. Epidemiological background data were available from the VBG. These data are not publicly available. The authors have no permission to make the data available. No administrative permissions were required to access the raw data. The VBG prepared and shared the specific files to the University Medical Center Wuerzburg. Data protection and confidentiality agreements were signed prior to study research.

## Abbreviations

VBG: Verwaltungs-Berufsgenossenschaft
PG: Point guard
SG: Shooting guard
SF: Small forward
PF: Power forward
C: Center
CI: Confidence interval
OR: Odds ratio
ACL: Anterior cruciate ligament
PCL: Posterior cruciate ligament
MCL: Medial collateral ligament
AC joint: Acromioclavicular joint

## References

[1] Drakos MC, Domb B, Starkey C (2010). Injury in the National Basketball Association: a 17-year overview. Sports Health.

[2] Bahr R, Krosshaug T (2005). Understanding injury mechanisms: a key component of preventing injuries in sport. Br J Sports Med.

[3] Finch C (2006). A new framework for research leading to sports injury prevention. J Sci Med Sport.

[4] van Mechelen W, Hlobil H, Kemper HC (1992). Incidence, severity, aetiology and prevention of sports injuries. A review of concepts. Sports Med.

[5] Luig P, Krutsch W, Henke T (2020). Contact: but not foul play—dominates injury mechanism in professional men's handball: a video match analysis of 580 injuries. Br J Sports Med.

[6] Klein C, Luig P, Henke T (2020). Nine typical injury patterns in German professional male football (soccer): a systematic visual video analysis of 345 match injuries. Br J Sports Med.

[7] Anderson T, Tenga A, Engebretsen L (2004). Video analysis of injuries and incidents in Norwegian professional football. Br J Sports Med.

[8] Arnason A, Tenga A, Engebretsen L (2004). A prospective video-based analysis of injury situations in Elite Male Football. Football incident analysis. Am J Sports Med.

[9] Oehlert K, Drescher W, Petersen W (2004). Injuries in Olympic handball tournaments: a video analysis. Sportverletz Sportschaden.

[10] Lemme NJ, Li YL, Kleiner JE (2019). Epidemiology and video analysis of achilles tendon ruptures in the National Basketball Association. Am J Sports Med.

[11] Panagiotakis E, Mok KM, Fong DTP (2017). Biomechanical analysis of ankle ligamentous sprain injury cases from televised basketball games: understanding when, how and why ligament failure occurs. J Sci Med Sport.

[12] Hewett TE, Torg JS, Boden BP (2009). Video analysis of trunk and knee motion during non-contact anterior cruciate ligament injury in female athletes: lateral trunk and knee abduction motion are combined components of the injury mechanism. Br J Sports Med.

[13] Koga H, Nakamae A, Shima Y (2010). Mechanisms for noncontact anterior cruciate ligament injuries. Knee joint kinematice in 10 injury situations from female team handball and basketball. Am J Sports Med.

[14] Krosshaug T, Nakamea A, Boden B (2007). Mechanisms of anterior cruciate ligament injury in basketball. Video analysis of 39 cases. Am J Sports Med.

[15] Koga H, Nakamae A, Shima Y (2018). Hip and ankle kinematics in noncontact anterior cruciate ligament injury situations: video analysis using model-based image matching. Am J Sports Med.

[16] Metz A. Entwicklung und Evaluation eines Beobachtungsinstruments zur systematischen Videoanalyse von Verletzungsereignissen im Profibasketball der Männer (Bachelor Thesis). Dt. Sporthochschule Köln, Cologne; 2015.

[17] Klein C, Henke T, Platen P (2018). Injuries in football (soccer)—a systematic review of epidemiology and aetiological aspects. Ger J Exerc Sport Res.

[18] Beaudouin F, aus der Funten K, Tross T (2020). Match situations leading to head injuries in professional male football (soccer)—a video-based analysis over 12 years. Clin J Sport Med.

[19] Dvorak J, Junge A, Grimm K (2007). Medical report from the 2006 FIFA World Cup Germany. Br J Sports Med.

[20] Cumps E, Verhagen E, Meeusen R (2007). Prospective epidemiological study of basketball injuries during one competitive season: ankle sprains and overuse knee injuries. J Sports Sci Med.

[21] Rodas G, Bove T, Caparros T (2019). Ankle sprain versus muscle strain injury in professional men’s basketball: a 9-year prospective follow-up study. Orthop J Sports Med.

[22] Takahashi S, Nagano Y, Ito W (2019). A retrospective study of mechanisms of anterior cruciate ligament injuries in high school basketball, handball, judo, soccer and volleyball. Medicine (Baltimore).

[23] Serner A, Mosler AB, Tol JL (2019). Mechanisms of acute adductor longus injuries in male football players: a systematic visual video analysis. Br J Sports Med.

[24] Beaudouin F, aus der Fünten K, Tröß T (2019). Head injuries in professional male football (soccer) over 13 years: 29% lower incidence rates after a rule change (red card). Br J Sports Med.

[25] Fuller CW, Junge A, Dvorak J (2005). A 6 year prospective study of the incidence and causes of head and neck injuries in international football. Br J Sports Med.

[26] Ryynänen J, Börjesson M, Karlsson J, Musahl V, Karlsson J, Krutsch W, Mandelbaum BR, Espregueira-Mendes J, d'Hooghe P (2018). Match-related factors influencing injury risk. Return to play in football: an evidence-based approach.

[27] Kofotolis N, Kellis E (2007). Ankle sprain injuries: a 2-year prospective cohort study in female Greek professional basketball players. J Athl Train.

[28] Meeuwisse WH, Sellmer R, Hagel BE (2003). Rates and risks of injury during Intercollegiate basketball. Am J Sports Med.

[29] Achenbach L, Krutsch V, Weber J (2018). Neuromuscular exercises prevent severe knee injury in adolescent team handball players. Knee Surg Sports Traumatol Arthrosc.

[30] Petushek EJ, Sugimoto D, Stoolmiller M (2018). Evidence-based best practice guidelines for preventing anterior cruciate ligament injuries in young female athletes: a systematic review and meta-analysis. Am J Sports Med.

[31] van Dyk N, Behan FP, Whiteley R (2019). Including the Nordic hamstring exercise in injury prevention programmes halves the rate of hamstring injuries: a systematic review and meta-analysis of 8459 athletes. Br J Sports Med.

[32] Askling C, Karlsson J, Thorstensson A (2003). Hamstring injury occurrence in elite soccer players after preseason strength training with eccentric overload. Scand J Med Sci Sports.

[33] Ishoi L, Sorensen CN, Kaae NM (2016). Large eccentric strength increase using the Copenhagen Adduction exercise in football: a randomized controlled trial. Scand J Med Sci Sports.

[34] Dalen-Lorentsen T, Bjoerneboe J, Clarsen B (2020). Does load management using the acute: chronic workload ratio prevent health problems? A cluster randomised trial of 482 elite youth footballers of both sexes. Br J Sports Med.

[35] Finch C (2003). How useful are insurance claim data for sports injury prevention purposes?. Injury Control Saf Promot..

[36] Åman M, Forssblad M, Henriksson-Larsén K (2014). Insurance claims data: a possible solution for a national sports injury surveillance system? An evaluation of data information against ASIDD and consensus statements on sports injury surveillance. BMJ Open.

[37] Henke T, Luig P, Schulz D (2014). Sports injuries in German club sports, aspects of epidemiology and prevention. Bundesgesundheitsblatt Gesundheitsforschung Gesundheitsschutz.

[38] Andersen TE, Tenga A, Engebretsen L (2004). Video analysis of injuries and incidents in Norwegian professional football. Br J Sports Med.

